# Effects of low-dose amiodarone and Betaloc on the treatment of hypertrophic cardiomyopathy complicated with malignant ventricular arrhythmias

**Published:** 2014

**Authors:** Yu Gao, Peisheng Zhang, Xue Liang

**Affiliations:** 1 Yu Gao, Second Department of Cardiology, The Fifth Affiliated Hospital of Zhengzhou University, Zhengzhou 450052, P. R. China.; 2 Peisheng Zhang, Second Department of Cardiology, The Fifth Affiliated Hospital of Zhengzhou University, Zhengzhou 450052, P. R. China.; 3 Xue Liang, Second Department of Cardiology, The Fifth Affiliated Hospital of Zhengzhou University, Zhengzhou 450052, P. R. China.

**Keywords:** Amiodarone, Betaloc, Hypertrophic cardiomyopathy, Malignant ventricular arrhythmias

## Abstract

***Objective:*** To study the therapeutic effects of low-dose amiodarone and Betaloc on hypertrophic cardiomyopathy complicated by malignant ventricular arrhythmias.

***Methods: ***Eighty-two such patients were selected and divided into a treatment group and a control group by the random number method (n=41), which were administered with low-dose amiodarone plus Betaloc and individual Betaloc respectively.

***Results:*** The treatment group had a significantly higher overall effective rate (85.4%) than the control group (65.9%) did. Based on the New York Heart Association's classification of cardiovascular disease, the treatment group mainly comprised Class III and IV patients before treatment, which were significantly relieved after treatment (P<0.05). The heart rate was evidently decreased from (119.99±18.91) bpm to (80.98±12.34) bpm, and the incidences of premature ventricular contraction and tachycardia were significantly reduced (P<0.05). The longest QT intervals after and before treatment were (421±32) ms and (411±35) ms respectively. The shortest QT interval after treatment [(350±36) ms] was significantly longer than that before [(307±31) ms]. The QT dispersion before treatment [(96±29) ms] was significantly higher that after [(64±17) ms] (P<0.05). Six out of eighty two patients in the treatment group succumbed to adverse reactions (14.63%).

***Conclusion: ***Hypertrophic cardiomyopathy complicated with malignant ventricular arrhythmias can be well treated with low-dose amiodarone and Betaloc, with mitigated symptoms, improved prognosis and few adverse reactions.

## INTRODUCTION

Hypertrophic cardiomyopathy, a common cardiovascular disease, is associated with gene mutation.^1^ With disease progression, patients suffer from fatigue, dyspnea, dizziness, and even sudden cardiac death.^2^ Particularly, the hypertrophic cardiomyopathy patients complicated with malignant ventricular arrhythmias are more prone to sudden cardiac death.^3^ Therefore, controlling the symptoms promotes the prognosis. Amiodarone, which is a Class III antiarrhythmic agent, functions by prolonging the effective refractory period of myocardial cells, decreasing the interval between action potentials, and eliminating reentry.^4^ Betaloc, a β-receptor blocker, can also treat arrhythmia by exerting antagonistic action on excited β-receptor through binding the ectopic pacemaker β1 adrenergic receptor of cardiomyocytes.^5^ Therefore, we herein aimed to study the effects of low-dose amiodarone in combination with Betaloc on hypertrophic cardiomyopathy complicated with malignant ventricular arrhythmias.

## METHODS

***General*** ***I*** ***nformation: ***All the studies were approved by the ethics committee of The Fifth Affiliated Hospital of Zhengzhou University, and consent was obtained from all patients. Eighty-two hypertrophic cardiomyopathy complicated with malignant ventricular arrhythmias patients who were treated in our hospital from March 2010 to March 2013 were selected, including 58 males and 24 females aged 24-53 years old, with the average age of (33.63±6.42) years old. All the patients suffered from sudden palpitation, dyspnea and chest tightness, and they were diagnosed as soon as possible after Holter examination, echocardiography and chest radiography. The patients were divided into a treatment group and a control group (n=41) by the random number method. The treatment group consisted of 29 males and 12 females aged 25-53 years old (average: 33.53±6.22), and the control group comprised 29 males and 12 females aged 24-53 years old (average: 33.73±6.62). The patients with ion disorder-induced arrhythmia, as well as abnormal liver, kidney and thyroid functions were excluded. There were no significant differences between the clinical features of the two groups (P>0.05).

***Inclusion*** ***C*** ***riteria: ***According to the Holter examination results and Lown's grading of premature ventricular contraction (PVC), only Grade 3 and above patients were selected in this study.^6^ "Guideline for the Diagnosis and Treatment of Hypertrophic Cardiomyopathy"^7^ was also used for patient selection. The patients with atrial fibrillation, hyperthyroidism and drug-induced arrhythmias were excluded.

***Treatment*** ***M*** ***ethods: ***The treatment group was orally administered with low-dose amiodarone and Betaloc ; Amiodarone: 0.2 g, bid; Betaloc: 6.25 mg, bid. After one week, the dose of amiodarone was decreased to 0.1 g (qd) and maintained thereafter. The dose of Betaloc was increased to 12.5 mg (bid) based on the patients' tolerance and maintained thereafter. The control group was only administered with Betaloc at the same dose. The patients were treated for a period of three months..

***Observation*** ***I*** ***ndices: ***The two groups were subjected to Holter examination before and after three treatment courses, during which they were also examined by routine electrocardiography. The longest and shortest QT intervals, QT dispersion, and adverse reaction events were observed.

***Evaluation*** ***S*** ***tandards for Therapeutic Effects*** ^8^ *: *The therapeutic effects were evaluated according to the Holter examination outcomes. Effective: Absence of sustained ventricular tachycardia, the incidence of non-sustained ventricular tachycardia was decreased by over 90%, and PVCs were decreased by over 75%. Ineffective: Presence of sustained and non-sustained ventricular tachycardia, and unrelieved PVCs.

***Statistical*** ***A*** ***nalysis: ***All data were analyzed by SPSS 17.0. The measurement data were expressed as mean ± standard deviation and compared by t test. P<0.05 was considered statistically significant.

## RESULTS

***Comparison between Therapeutic Effects: ***The overall effective rate of the treatment group (85.4%) was significantly higher than that of the control group (65.9%) (P<0.05) (Table-I).

***Liver, Kidney, and Cardiac Functions before and after*** ***T*** ***reatment: ***The liver and kidney functions of patients did not differ significantly before and after treatment (P>0.05). The NYHA Class III and IV patients were significantly decreased after treatment (P<0.05). Besides, the heart rate was significantly reduced from (119.99±18.91) bpm to (80.98±12.34) bpm (P<0.05). Moreover, the incidences of PVC and tachycardia were also significantly lowered (P<0.05) (Table-II).

***Longest,*** ***S*** ***hortest QT*** ***I*** ***ntervals and*** ***Q*** ***T*** ***D*** ***ispersion before and after*** ***T*** ***reatment: ***The longest QT intervals after and before treatment were (421±32) ms and (411±35) ms respectively. The shortest QT interval after treatment [(350±36) ms] was significantly longer than that before [(307±31) ms]. The QT dispersion before treatment [(96±29) ms] was significantly higher that after [(64±17) ms] (P<0.05) (Table-III).

***Adverse*** ***R*** ***eactions of the*** ***T*** ***reatment*** ***G*** ***roup: ***Four patients in the treatment group were prone to nausea and vomiting which were mitigated without influencing the outcomes. Two patients who suffered from sinus bradycardia claimed tolerance, thus being further treated without special care. The incidence of adverse reactions was 14.63% (6/41).

## DISCUSSION

Hypertrophic cardiomyopathy, a cardiovascular disease, is related with the mutation of gene-encoding cardiac sarcomeric genes.^9^ The patients are clinically manifested as differently thicked cardiac muscle fibers that results from asymmetric ventricular hypertrophy and disorderedly arranged myocardial cells. Therefore, reentrant excitation is induced owing to different pathways of cardiac electrophysiological conduction. Reentrant excitation may give rise to various ventricular arrhythmia symptoms. With disease progression, patients may die of sudden cardiac death due to hemodynamic instability.^10^ Especially, 0.1%-1.0% of the patients may die without early signs.^11^ It has previously been reported that malignant arrhythmia was directly associated with sudden cardiac death.^12^^,^^13^

**Table-I:** Comparison between therapeutic effects

*Group*	*Case No.*	*Markedly effective*	*Effective*	*Ineffective*	*Overall effective rate*
Treatment group	41	24	11	6	85.4^a^
Control group	41	14	13	14	65.9

Compared with the control group,

a P<0.05.

**Table-II:** Liver, kidney, and cardiac functions before and after treatment

*Item*	*Case No.*	*Before*		*After*				*t/χ* ^2^	*P*
		*Class III*	*Class IV*	*Class I*	*Class II*	*Class III*	*Class IV*		
NYHA classification	40	27	13	3	25	9	3	5.6721	<0.05
Heart rate (bpm)	40	119.99±18.91		80.98±12.34				10.9265	<0.05
PVC	40	34 (85)		9 (22.5)				31.4268	<0.05
Tachycardia	40	14 (35)		3 (7.5)				9.0383	<0.05

**Table-III:** Longest, shortest QT intervals and QT dispersion before and after treatment

*Index*	*Before*	*After*
Longest QT interval	411±35	421±32
Shortest QT interval	307±31	350±36^a^
QT dispersion	96±29	64±17^a^

Compared with the results before treatment,

a P＜0.05.

 Amiodarone, as a Class III antiarrhythmic agent, can prolong the effective refractory period of myocardial cells, shorten the interval between action potentials, decelerate conduction and terminate reentrant excitation by non-competitively binding α- and β-adrenergic receptors on the myocardial cell membrane.^14^ In this study, the patients were also well treated with amiodarone.

Betaloc, which is a β-receptor blocker, can clinically treat hypertension patients who have fast heart rates. Betaloc effectively stabilizes ventricular rate by binding β1 adrenergic receptor, exerting antagonistic effects on its excitation, suppressing sympathetic excitement, decreasing the phase-4 depolarization rate and phase-0 action potential rising rate of cardiomyocytes, as well as reducing their self-regulation and conduction rate.^15^ Meanwhile, Betaloc can inhibit the proliferation and overoxidation of myocardial cells, prevent them from apoptosis, reverse cardiac remodeling, and alleviate ventricular hypertrophy.^16^ Hence, Betaloc not only facilitates the control of ventricular rate and arrhythmia, but also promotes the reversion of hypertrophic cardiomyopathy. In this study, low-dose amiodarone in combination with Betaloc treated hypertrophic cardiomyopathy complicated with malignant ventricular arrhythmias effectively and safely.

QT interval is a measure of the time between the start of the Q wave and the end of the T wave in the electrical cycle of heart. QT dispersion, i.e. maximum QT interval minus minimum QT interval, is a non-invasive index reflecting the instability of electrical activity in ventricular myocytes and the inhomogeneity of repolarization.^17^ In this study, the longest QT interval was not significantly changed after treatment, while the shortest QT interval was significantly prolonged and QT dispersion was dramatically shortened, Since QT dispersion is a predicting factor of ventricular arrhythmia and sudden cardiac death,^18^ the shortened one in this study suggested satisfactory therapeutic effects and improved prognosis.

In summary, low-dose amiodarone plus Betaloc can treat hypertrophic cardiomyopathy complicated with malignant ventricular arrhythmias with excellent outcomes, improved prognosis and few adverse reactions, thus being worthy of wider application.
